# Community health workers: A sustainable health system innovation or just an emergency response?

**DOI:** 10.3389/fpubh.2022.1040539

**Published:** 2022-12-06

**Authors:** Caroline Masquillier, Theo Cosaert

**Affiliations:** ^1^Department of Sociology, University of Antwerp, Antwerp, Belgium; ^2^Department of Family Medicine and Population Health, University of Antwerp, Antwerp, Belgium; ^3^Department of Public Health, Institute of Tropical Medicine, Antwerp, Belgium

**Keywords:** community health workers, strengthening health systems, strengthening health workforce, emergency response, innovation

## Introduction

The detrimental effects of the COVID-19 pandemic on people's health and health systems worldwide are readily apparent (1). Even in well-resourced and well-performing health systems, this public health crisis has further exposed existing weaknesses—such as inequitable access to healthcare for socio-economically vulnerable groups, shortages of health personnel, and absence of rapid local interventions for prevention, health promotion and vaccination (1–3). The COVID-19 pandemic has made clear that health workers are needed on the ground to provide quick local responses (2, 3) and ensure access to care for all, including socio-economically vulnerable groups who are often left behind (4).

Early in the COVID-19 pandemic, community health workers (CHWs) became more widely recognized for their potential as an effective frontline response and capacity to improve access to care for populations living in socially vulnerable conditions (5). CHWs “are frontline public health workers who are trusted members of and/or have an unusually close understanding of the community served. This trusting relationship enables CHWs to serve as a liaison/link/intermediary between health/social services and the community to facilitate access to services and improve the quality and cultural competence of service delivery” (6). As such, CHWs provide links to the healthcare system, deliver interventions at the individual and family levels, play a role at the community level regarding actions on social determinants of health and signal structural shortcomings in healthcare system to inform policy making (7–12). In countries with established CHW-programmes, CHWs were quick to respond to the pandemic at the local level to maintain community trust, provide clear and straightforward information, explain prevention measures and establish a contact tracing system (13).

## COVID-19 pandemic as catalyst for innovation: Introduction of CHWs in Belgium

Whilst having detrimental effects for many populations living in socially vulnerable conditions (14), the COVID-19 pandemic also stimulated health system innovations (1). Inspired by CHW programmes in low-and-middle income countries (LMICs), researchers advocated for improved and expanded implementation of CHW programmes in high-income countries (HICs) (9, 15, 16). In the United States (US), for example, President Biden has called for the hiring of 100,000 additional CHWs as part of his strategy against the COVID-19 pandemic, building on the long-standing CHW initiatives in this country since their advent in the 1950s (5, 6). While CHW programs exist in some HICs – such as Canada (17), Australia (18), and New Zealand (19), among others—a scoping review concludes that CHWs in HICs “are an under-recognized, and therefore underutilized, public health workforce, which has a promising capacity to reduce health inequities in marginalized populations” ((17), p. e157).

Tapping into this potential, in response to the COVID-19 pandemic which exacerbated the access to care challenges for people living in socio-economic vulnerable circumstances, CHWs were introduced throughout Belgium at the start of 2021. More specifically, the Belgian Federal Government gave the National Institute for Sickness and Disability Insurance (NIHDI) and the National InterMutualist College (Intermut) the task to employ 50 CHWs in socio-economically vulnerable neighborhoods in ten Belgian cities. The CHWs followed a basic training and receive renumeration for their work. The CHWs' task is to improve accessibility to primary health care for people living in socially vulnerable conditions. The results of this first year are promising. A qualitative study shows that CHWs in the Belgian Federal project reach people who live at the intersection of different vulnerabilities, which are intertwined and can be mutually reinforcing. The CHWs consider the various barriers a person experiences when they provide them with support. The qualitative research concludes that CHWs can make an important contribution to improving access to care for people living in socially vulnerable circumstances, by tailoring their support to the needs of the target population and by signaling shortages in the healthcare system (20).

The Belgian CHW project emerged in a context without long-term funding or a sustainable policy vision on the issue (20). The budget was initially allocated for only one project year. It was renewed for a second year by the end of 2021. Experiences of CHW programmes in the past in other contexts allow us to learn what works today (2). We should not forget the disappointments of the programmes in the 1970s and 1980s in response to the Alma Ata Conference and the push for primary health care for all (10, 21). These taught us that CHW programmes are complex and a solid support system is needed to strengthen the CHW workforce through capacity building, systems readiness, CHWs' inclusion in all steps of the development and evaluation of CHW-projects and CHW credentialing opportunities, among others. In addition, to realize the full potential of CHWs, a long-term vision and sustainable funding is required (5, 22, 23). While functional CHW programmes are not cheap (22), they are proven to be cost-effective in reaching people living in socially vulnerable conditions (3). We should see CHW programmes not as a “temporary and underfunded afterthought,” but as “an integral component of optimally functioning health systems” ((3), p. 14). Our study of the Belgian CHW project emphasizes this notion (20). To ensure that every person receives the health services they need, it is important that CHWs are linked to the broader health system (2). Setting up collaboration with actors in healthcare takes time, as well as planning and coordination (24). The qualitative results showed that in the Belgian Federal project, links between CHWs and the health system mostly emerged on an ad-hoc and individual basis. Lack of sustainable funding and a long-term vision among Belgian policy makers and funders inhibits integration and synchronization of the federal CHW programme with the Belgian health system (20).

## Discussion

As we enter a new stage of this COVID-19 public health crisis, we need to seize the moment to build resilient health systems fit for the future (1). It is time to move beyond emergency response pilot projects in countries like Belgium. We should implement a CHW model that plays an integral role in the health system, following the example of countries like Brazil. Emerging as a pilot project amidst serious droughts in the 1980s, the Brazilian CHW programme is now one of the exemplary CHW models (2). In the Brazilian Family Health Strategy, primary care is provided by a Family health team—consisting of a physician, a nurse, a nurse assistant, and four to six full-time CHWs. Each team interacts with all households in a geographically defined community (25, 26). At least once a month, irrespective of need or demand, the CHW visits each household proactively in their micro-area. CHWs focus on all members of the household with a package of health-promotion activities that fully spans the life course (27). Other well integrated CHW programs into national public health care systems include those in Ethiopia, Bangladesh and Nepal, among others (10).

In a larger health system reform, CHWs fill a void in healthcare provision in a unique way by providing quick local responses and ensuring access to care for all (5). The launch of the Belgian CHW project demonstrated that there is political support for this innovative healthcare model. What we need now is to mobilize continued political will (2) to sustain the lessons learnt in times of crisis in times of plenty (16). It is time to endorse the future of CHWs and equitable and accessible healthcare for all, by looking beyond emergency responses, by strengthening this CHW workforce, by formulating a long-term vision and by ensuring sustainable funding for health system innovations set in motion by the COVID-19 pandemic.

## Author contributions

Conceptualization, writing—original draft preparation, and funding acquisition: CM. Writing—review and editing: CM and TC. All authors contributed to the article and approved the submitted version.

## Conflict of interest

The authors declare that the research was conducted in the absence of any commercial or financial relationships that could be construed as a potential conflict of interest.

## Publisher's note

All claims expressed in this article are solely those of the authors and do not necessarily represent those of their affiliated organizations, or those of the publisher, the editors and the reviewers. Any product that may be evaluated in this article, or claim that may be made by its manufacturer, is not guaranteed or endorsed by the publisher.
